# Minicircle DNA Provides Enhanced and Prolonged Transgene Expression Following Airway Gene Transfer

**DOI:** 10.1038/srep23125

**Published:** 2016-03-15

**Authors:** Mustafa M. Munye, Aristides D. Tagalakis, Josephine L. Barnes, Rachel E. Brown, Robin J. McAnulty, Steven J. Howe, Stephen L. Hart

**Affiliations:** 1 UCL Institute of Child Health, 30 Guilford Street, London, WC1N 1EH, United Kingdom; 2 UCL Respiratory Centre for Inflammation and Tissue Repair, 5 University Street, London, WC1E 6JF, United Kingdom; 3 UCL MRC Laboratory for Molecular Cell Biology, Gower Street, London WC1E 6BT, United Kingdom

## Abstract

Gene therapy for cystic fibrosis using non-viral, plasmid-based formulations has been the subject of intensive research for over two decades but a clinically viable product has yet to materialise in large part due to inefficient transgene expression. Minicircle DNA give enhanced and more persistent transgene expression compared to plasmid DNA in a number of organ systems but has not been assessed in the lung. In this study we compared minicircle DNA with plasmid DNA in transfections of airway epithelial cells. *In vitro*, luciferase gene expression from minicircles was 5–10-fold higher than with plasmid DNA. In eGFP transfections *in vitro* both the mean fluorescence intensity and percentage of cells transfected was 2–4-fold higher with minicircle DNA. Administration of equimolar amounts of DNA to mouse lungs resulted in a reduced inflammatory response and more persistent transgene expression, with luciferase activity persisting for 2 weeks from minicircle DNA compared to plasmid formulations. Transfection of equal mass amounts of DNA in mouse lungs resulted in a 6-fold increase in transgene expression in addition to more persistent transgene expression. Our findings have clear implications for gene therapy of airway disorders where plasmid DNA transfections have so far proven inefficient in clinical trials.

Lung diseases are a major cause of morbidity including genetic diseases, such as cystic fibrosis (CF), primary ciliary dyskinesia (PCD), surfactant protein B (SPB)-deficiency and alpha 1-antitrypsin (A1AT) –deficiency^1^. The lung is an attractive organ for gene therapy of these diseases given its accessibility allowing for vector delivery by inhalation. CF is the most common respiratory genetic disease and, since the cystic fibrosis transmembrane conductance regulator gene (*CFTR*) was identified in 1989^2^, it has become one of the most intensely studied diseases in the field of gene therapy^1^,^3^. A number of human clinical trials have been conducted^4^,^5^,^6^,^7^,^8^,^9^,^10^,^11^ but clinically viable gene therapy treatments for CF or other genetic lung disorders have yet to materialise. This lack of clinical efficacy has been in large part due to inefficient transgene expression resulting from the mucociliary barriers that the respiratory system has evolved to clear inhaled foreign particles.

More recently, PCD has been proposed as a candidate respiratory disease for treatment by gene therapy. PCD, like CF, affects the ciliated airways from the bronchi to the bronchioles and thus faces many of the same challenges in achieving therapeutic levels of lung transfection, although it is genetically far more heterogeneous than CF with 30 disease-causing genes identified to date^12^. Mutations in the *DNAH5* and *DNAI1* genes, which both code for outer dynein arm proteins essential for normal cilia motility, are the most common cause of PCD together accounting for around 30% of patients^13^,^14^. Proof of concept for PCD gene therapy has been demonstrated *in vitro* in human cells from patients with mutations in the *DNAI1* gene^15^ and murine cells from a mouse model of PCD where the gene for the outer dynein arm protein *Dnaic1* (murine homolog of human DNAI1) was mutated^16^.

Gene therapy for lung diseases will likely require repeated administration several times a year for the lifetime of the patient given the limited lifespan of airway epithelial cells. Therefore, non-viral gene delivery formulations are best suited for this application owing to their low toxicity and low immunogenicity allowing repeated dosing. In addition, non-viral formulations can be delivered by nebulisation and may be produced at a large-scale to clinical grade. However, as noted in clinical trials of CF gene therapy, with liposomal and polymeric formulations, clinically relevant levels of gene transfer have proven difficult to achieve.

We have focused our recent research on an improved nanocomplex formulation of lipids and peptides in which these components interact synergistically to enhance transfection efficiency^17^. We have developed nanocomplex formulations targeted to the airway epithlium and found a nanocomplex termed LED-1 efficiently transfected airway epithelial cells *in vivo*^18^. LED-1 is formulated with a cationic **L**iposome made up of DHDTMA/DOPE lipids, a cationic peptide with residues K_16_GACSERSMNFCG (referred to as peptide**E**) and plasmid **D**NA. The nanocomplexes are targeted to the airway epithelial cells via peptide ligands^18^,^19^ while the lipid components help to overcome endosomal barriers to transfection producing greatly improved levels of epithelial specific transfection *in vitro* and *in vivo*^20^. Towards developing optimal formulations and protocols for airway epithelial gene transfer, we then optimised the methods of nebulised delivery to the conducting airways in mice^21^ and pigs^22^.

In addition to improving the delivery formulation, we are now focusing on optimisation of the DNA payload itself. Elements of the plasmid DNA payload that have been optimised to either enhance or prolong transgene expression have included the promoter, enhancer elements and polyadenylation signals as well as the removal of all CpG motifs to reduce inflammation^23^,^24^,^25^,^26^,^27^. However, plasmids also contain a bacterial backbone consisting of prokaryotic replication origin sequences and antibiotic resistance genes to allow for easy propagation and purification of plasmid DNA which are not necessary for the gene therapy formulation.

Recently a number of groups have removed the bacterial backbone from plasmid DNA to produce minicircle DNA by adding recombination sequences between the transgene expression cassette and the bacterial backbone, followed by recombination in *E.coli* to produce minicircles^28^,^29^,^30^. Several studies have shown that minicircle DNA provide enhanced transgene expression in a variety of organ systems *in vivo* including the liver^31^,^32^,^33^, heart^34^,^35^ and skeletal muscle^35^ but minicircles have not been assessed for airway gene delivery. In this study we have produced a number of minicircles containing reporter transgenes and the 14 kb *DNAH5* cDNA encoding an outer dynein arm protein involved in PCD, the largest minicircle produced to date. We assessed whether minicircles could enhance transgene expression in airway epithelial cells *in vitro* and *in vivo* in mice. Furthermore, we have assessed the persistence of transgene expression *in vivo*, and assessed the inflammatory response to minicircle and plasmid DNA transfections.

## Materials and Methods

### Materials

Peptide K_16_GACSERSMNFCG^19^ was synthesised by China Peptide Co., Ltd (Shanghai, China), and dissolved in endotoxin-free water (Sigma-Aldrich, Dorset, UK) to 10 mg/mL. Liposome consisted of 1-Propanaminium, *N,N,N*-trimethyl-2,3-bis(11*Z*-hexadecenyloxy)-chloride (DHDTMA chloride; Avanti Polar Lipids; Alabama, USA) with dioleoylphosphatidylethanolamine (DOPE; Avanti Polar Lipids; Alabama, USA) formulated at a 1:1 weight ratio and dissolved in sterile water to give a 2 mg/mL liposome suspension.

### Cloning and minicircle production

**Figure 1:**
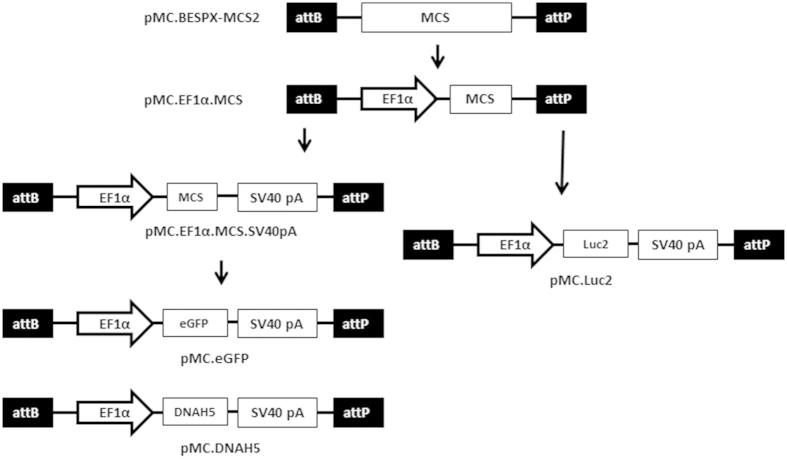
Schematic of the cloning of minicircle producer plasmids. Minicircle producer plasmids coding for eGFP (pMC.eGFP), DNAH5 (pMC.DNAH5) and firefly luciferase (pMC.Luc2) were produced by sub-cloning, sequentially, the EF1α promoter, SV40 polyadenylation signal and transgene cDNA sequences into the empty minicircle producer plasmid pMC.BESPX-MCS2.

Minicircle producer plasmids expressing enhanced green fluorescent protein (eGFP), firefly luciferase (Luc2), and DNAH5 were produced by cloning into the commercial plasmid pMC.BESPX-MCS2 (Cambridge Biosciences; Fig. 1). To do this, the EF1α promoter from pCpGfree-LacZ (Invivogen, Toulouse, France) was cloned into pMC.BESPX-MCS2 and Luc2 gene and SV40 polyadenylation signal from pGL4.10 (Promega, Southampton, UK) sub-cloned to produce pMC.Luc2. Alternatively, SV40 polyadenylation signal from pCpGfree-LacZ was sub-cloned into pMC.EF1α.MCS and eGFP cDNA from pEGFP-N1 (Clontech, Saint-Germain-en-Laye, France) sub-cloned to produce pMC.eGFP. DNAH5 cDNA was PCR amplified from reverse transcribed RNA (Omniscript RT kit; Qiagen, Crawley, UK) extracted from ciliated human airway epithelial cells. Given the size of *DNAH5,* the full-length cDNA was cloned as three overlapping segments each of which were TOPO cloned into pCR-XL-TOPO plasmid (Life Technologies, Paisley, UK). The cDNA was flanked with *KpnI* and *SalI* sites and naturally occurring *AfeI* and *SexAI* sites were utilised to reconstruct the full-length DNAH5 cDNA which was subsequently sub-cloned into pMC.EF1α.MCS.SV40pA to produce pMC.DNAH5. Minicircle DNA (MC) was produced from the minicircle producer plasmid (pMC) as previously described^30^ and agarose gel electrophoresis showed no detectable plasmid contamination in minicircle DNA preparations (Figure S1).

### Cell culture

16HBE14o- cells (kind gift from Professor Dieter Gruenert, San Francisco^36^) were cultured in complete media consisting of Eagle’s Minimal Essential Medium (MEM; Sigma-Aldrich, Dorset, UK) supplemented with 10% (v/v) foetal bovine serum, 100 U/mL penicillin, 100 mg/mL streptomycin, 2 mmol/l L-glutamine and incubated in humidified 5% CO_2_ at 37 °C.

### *In vitro* transfections

Cells were seeded in 96-well plates at a density of 20,000 cells per well and incubated in humidified 5% CO_2_ at 37 °C for 24 hours. For luciferase assay experiments, cells were seeded in black plates with clear bottoms (Fisher Scientific UK, Leicestershire, UK). All complexes were prepared in OptiMEM (Life Technologies, Paisley, UK). LED-1 complexes were prepared at a 0.75:4:1 weight ratio of liposome: peptide: plasmid/minicircle DNA as optimised previously^18^. Complexes were formulated to a plasmid/minicircle DNA concentration of 10 μg/mL and prepared by first mixing the cationic liposome and peptide solutions followed by addition of the DNA solution. Formulations were incubated at room temperature for 30 minutes to allow for complex self-assembly and 25 μL (250 ng for plasmid DNA or equal mass minicircle DNA) added to cells in 175 μL complete media, centrifuged at 378 × *g* for 5 minutes and incubated in humidified 5% CO_2_ at 37 °C for 24 or 48 hours for luciferase and flow cytometry assays, respectively. Where appropriate, complexes were diluted in OptiMEM to equimolar amounts of DNA before incubating with cells.

### Luciferase assay following *in vitro* transfection

Cells were washed with 1x PBS 24 hours following transfection and lysed with 20 μL Reporter Lysis Buffer (Promega, Southampton, UK) for 20 minutes at 4 °C then −80 °C for at least 30 minutes followed by thawing at room temperature. Luciferase activity was assessed using the Luciferase Assay System (Promega, Southampton, UK) on a FLUOstar Optima plate reader (BMG Labtech, Aylesbury, UK). The results were standardised for protein content using the Bradford protein assay (Bio-Rad, Hertfordshire, UK) and expressed as relative luminescence units per mg of protein (RLU/mg). Assessment of luciferase activity at 24 hours was chosen as firefly luciferase is known to have a short half-life (3h)^37^ and expression following lung gene delivery *in vivo* has been shown to be maximal at 24 hours^38^,^39^.

### Flow cytometry

Adherent cells in 96-well plates were detached using 50 μL Trypsin-EDTA (Sigma-Aldrich, Dorset, UK) and re-suspended with 150 μL DPBS (Sigma-Aldrich, Dorset, UK). Cells were then acquired with a BD FACSArray flow cytometer (BD Biosciences, Oxford, UK) and analysis was performed with FlowJo software v. 8.8.3 (Tree Star Inc., Olten, Switzerland).

### qRT-PCR

Cells were harvested 48 hours following transfection and homogenised with Qiagen shredders (Qiagen, Crawley, UK). Total RNA was isolated from the homogenate using a Qiagen RNeasy Mini kit (Qiagen, Crawley, UK) and DNAse digested (TURBO DNAse; Life Technologies, Paisley, UK). cDNA was then synthesised with 2 μg of total RNA as the template for Omniscript reverse transcriptase (Omniscript RT kit; Qiagen, Crawley, UK) in a 1 hour reaction at 37 °C and subsequently used in a qPCR reaction containing Platinum Quantitative PCR SuperMix-UDG w/ROX (Life Technologies, Paisley, UK) and TaqMan Gene Expression Assay primers and probe (Assay ID- DNAH5, Hs00292485_m1). Real-time quantitative PCR was performed using an ABI PRISM 7000 Sequence Detection System. The PCR reaction cycles used were 50 °C for 2 minutes, 95 °C for 10 minutes, 40 cycles of 95 °C for 15 seconds and 60 °C for 1 minute. Fluorescence data was collected at the end of each 60 °C reaction and relative expression levels calculated using the delta-delta Ct (2^−ΔΔCt^) method^40^.

For qPCR of plasmid and minicircle DNA, cells were harvested 48 hours following transfection and DNA extracted with DNeasy Blood & Tissue Kit (Qiagen, Crawley, UK). Purified DNA was then used in a qPCR reaction as described above for synthesised cDNA.

### *In vivo* transfections

All procedures were approved by UCL animal care policies and were carried out under Home Office Licenses issued in accordance with the United Kingdom Animals (Scientific Procedures) Act 1986 (UK). 5–6 week-old immunocompetent female CD1 mice (Charles River, Margate, UK) were used for all *in vivo* experiments. Complexes were prepared as described for *in vitro* transfections except complexes were made in pyrogen free water (Baxter, Berkshire, UK) to a final DNA concentration of 333.3 μg/mL and 50 μL (16.7 μg for plasmid DNA or equal mass minicircle DNA) was given by oropharyngeal instillation following gaseous isoflurane induced anaesthesia. Where appropriate, complexes were diluted in pyrogen free water to equimolar amounts of DNA before oropharyngeal instillation.

### Luciferase assay following *in vivo* transfection

24 hours, 7 days or 14 days following oropharyngeal instillation of nanocomplexes mice were culled and their lungs extracted and snap frozen. Lungs were weighed and immersed in 400 μL of ice cold reporter lysis buffer (Promega, Southampton, UK) per 100 mg of tissue and homogenised on ice using a T 10 basic Ultra Turrax homogeniser (IKA, Staufen, Germany) and then centrifuged at 14,170 × g for 10minutes at 4 °C. The supernatant was transferred to an autoclaved microfuge tube and centrifuged for a further 10 minutes at 4 °C and luciferase activity was assessed using the Luciferase Assay System (Promega, Southampton, UK) as described above. Results were expressed as relative luminescence units per milligram of protein (RLU/mg).

### Histology

24 hours following transfection, mouse lungs were inflation-fixed by intratracheal instillation of 4% (w/v) paraformaldehyde in PBS post-mortem and extracted lungs processed and stained with haematoxylin and eosin to demonstrate tissue architecture and inflammatory cell infiltration as previously described^41^.

### Bronchoalveolar lavage fluid analysis

24 hours following transfection, mice were culled and bronchoalveolar lavage fluid collected and processed for cytokine and chemokine quantification as previously described^42^. The presence of inflammatory cytokines in BALF was assessed and quantified using the Cytokine Mouse 10-Plex Panel (Life Technologies, Paisley, UK) and plates were read using a Bio-Plex 100 reader (Bio-Rad, Hertfordshire, UK) and data collected with Bio-Plex Manager software (v 4.1.1; Bio-Rad, Hertfordshire, UK).

### Statistical analysis

Data are mean values ± standard error of the mean (SEM) for *in vitro* studies and median with all data points shown for *in vivo* studies. For *in vitro* studies Student’s t-test was used to assess statistical significance. For *in vivo* studies where normal distribution was not observed non-parametric Kruskal-Wallis test followed by Dunn’s multiple comparison test was used to assess statistical significance. Significance levels were set at p < 0.05. *p < 0.05; **p < 0.01; ***p < 0.001; NS = not significant.

## Results

### Minicircle DNA production

Minicircle DNA (MC) was produced from minicircle producer plasmids (pMC) as previously described^30^. To assess the purity of minicircle DNA 500 ng of both plasmid and minicircle DNA preparations were digested with single cutting restriction enzymes and electrophoresed on a 1% ethidium bromide stained agarose gel and visualised under UV light. We found no detectable plasmid contamination in minicircle DNA preparations suggesting efficient minicircle production (Figure S1).

### *In vitro* transfections

**Figure 2:**
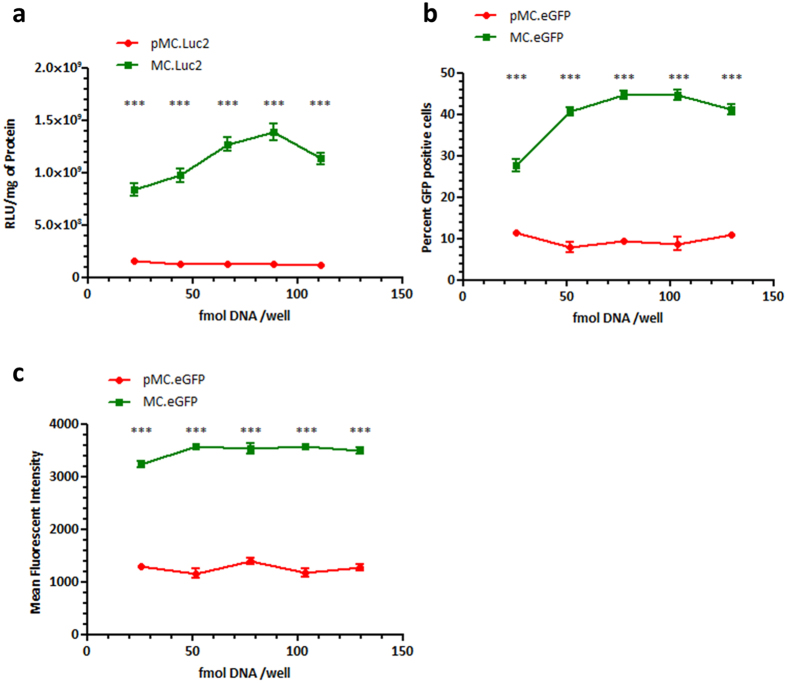
Transgene expression following *in vitro* transfections. 16HBE14o- cells were transfected with minicircle DNA (green line) or plasmid DNA (red line) carrying the firefly luciferase gene (**a**) or the eGFP gene (**b,c**). For eGFP transfections both the percentage of positive cells (**b**) and the MFI (**c**) were assessed. ***P < 0.01; Student’s t-test used to assess significance. Values are background subtracted and displayed as mean ± SEM.

16HBE14o- cells^36^ were used for *in vitro* studies as a representative cell type of the bronchial epithelium, the target for gene therapy of CF and PCD. Cells were transfected using a non-viral vector (LED-1) previously optimised for airway gene delivery to mammalian cells *in vitro* and *in vivo*^18^. Cells were transfected with varying amounts of minicircle and plasmid DNA carrying either the firefly luciferase gene (MC.Luc2 and pMC.Luc2, respectively) or the eGFP gene (MC.eGFP and pMC.eGFP, respectively). Cells transfected with minicircle DNA showed 5–10-fold higher levels of luciferase activity than plasmid DNA (Fig. 2a; p < 0.001) at equivalent doses. Flow cytometry analysis of eGFP transfected cells revealed a 2.5 to 4-fold increase in the percentage of cells expressing eGFP after transfection with minicircle DNA compared to plasmid DNA (Fig. 2b; p < 0.001 with all doses tested). Furthermore, the mean fluorescent intensity of cells transfected with eGFP minicircle DNA was 2.5 to 3-fold higher than cells transfected with plasmid DNA (Fig. 2c; p < 0.001with all doses tested). Fluorescence microscopy observations provided visual confirmation of the flow cytometry data (Figure S2).

Encouragingly, when the commercial transfection reagents Lipofecatmine 2000 and 25 kDa branched polyethylenimine (PEI) were used to transfect 16HBE14o- cells minicircle DNA showed 3–6-fold higher levels of luciferase activity than plasmid DNA (Figure S3; p < 0.001).

**Figure 3:**
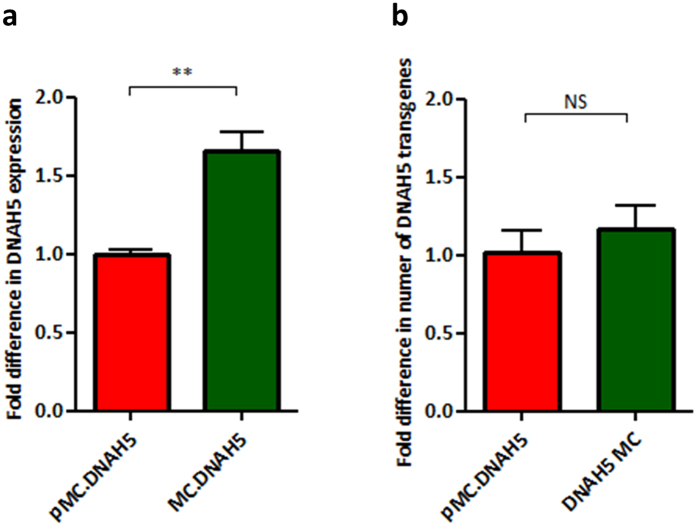
DNAH5 expression following *in vitro* minicircle and plasmid DNA transfections. 16HBE14o- cells were transfected with pMC.DNAH5 or MC.DNAH5 complexed into LED-1 particles and the relative amounts of DNAH5 (**a**) mRNA and (**b**) vector specific DNA quantified using qPCR and the 2- ΔΔCt method. NS, not significant; **P < 0.01; Student’s t-test used to assess significance. Values are mean ± SEM.

Using the LED-1 formulation, transfection of the 14 kb *DNAH5* cDNA, which encodes a protein in the ciliary outer dynein arm, resulted in 66.0 ± 11.8% higher levels of vector specific mRNA expression from the minicircle MC.DNAH5 than the plasmid pMC.DNAH5 (Fig. 3a; p < 0.01) when the same number of DNA molecules was transfected into 16HBE14o- cells.

We have previously shown a positive correlation between DNA size and the diameter of liposome:peptide:DNA nanoparticles^43^. The diameter of nanoparticles is known to affect their cellular uptake^44^ potentially affecting transgene expression. With *DNAH5* coding DNA the plasmid was 19.1 kb compared to 15.1 kb for the minicircle and we found nanoparticles formulated with pMC.DNAH5 plasmid had a diameter of 92.9 ± 0.6 nm compared to 110.5 ± 1.1 nm for LED-1 formulated with MC.DNAH5 minicircle DNA (Figure S4; p < 0.001). Both formulations yielded nanoparticles with a highly positive surface charge of 39.0 ± 0.4 mV and 35.4 ± 0.5 mV respectively (Figure S4; p < 0.01). Similarly, LED-1 complexes formulated with firefly luciferase and eGFP coding plasmid and minicircle DNA were around 100 nm and had a highly positive surface charge of between 35–40 mV (Figure S4). Whilst statistically significant these small differences should not have affected the uptake of LED-1 particles, and therefore transfection efficiency, given that the sizes were well below the 200 nm upper limit for clathrin mediated endocytosis^44^. Supporting this hypothesis, using qPCR analysis of DNA extracted from transfected 16HBE14o- cells we confirmed that equal numbers of plasmid and minicircle DNA molecules were found in 16HBE14o- cells transfected with equimolar amounts of pMC.DNAH5 plasmid and MC.DNAH5 minicircles (Fig. 3b).

### *In vivo* transfections

**Figure 4:**
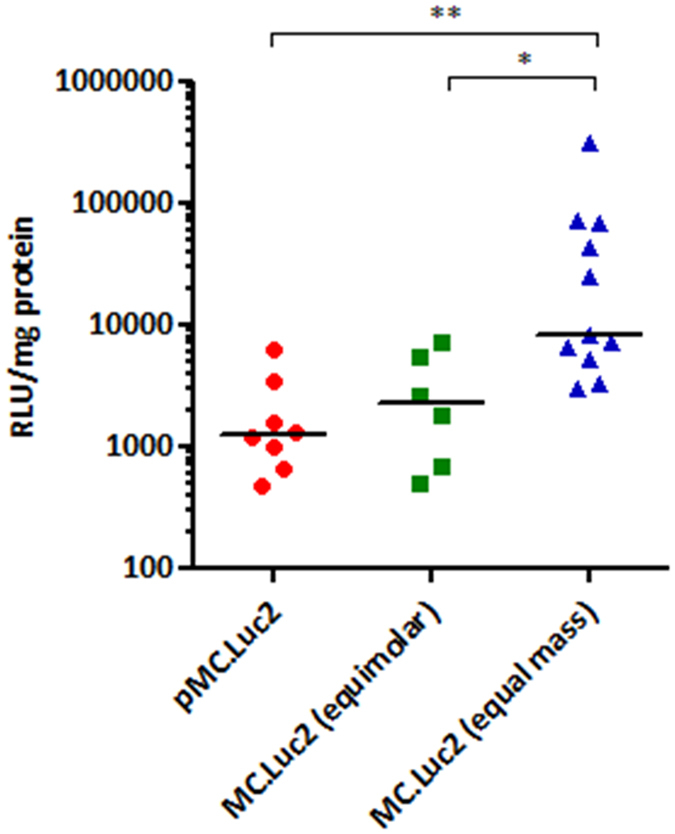
Transgene expression following *in vivo* minicircle and plasmid DNA transfections. Luciferase activity 24 hours following oropharyngeal instillation of CD1 mice with LED-1 complexes containing pMC.Luc2 plasmid DNA or MC.Luc2 minicircle DNA at either equimolar or equal mass quantities. *P < 0.05; **P < 0.01; Kruskal-Wallis test followed by Dunn’s post-test analysis to assess significance. Values are background subtracted and bar represents median RLU/mg.

Next we compared reporter gene expression from minicircle and plasmid DNA *in vivo*. Murine lungs were transfected by oropharyngeal instillation with a single dose of LED-1 complexes formulated with 16.7 μg of plasmid DNA (pMC.Luc2) or minicircle DNA (MC.Luc2). The lungs were harvested 24 hours later and lung extracts assayed for luciferase activity. Minicircle DNA gave a 6.5-fold higher median level of transgene expression than plasmid DNA (Fig. 4; median value of 8115.9 RLU/mg for minicircle DNA and 1249 RLU/mg for plasmid DNA; p < 0.01). However, in contrast to *in vitro* transfections, there was no significant difference in transfection efficiency using equimolar amounts of plasmid (16.7 μg) and minicircle DNA (7.1 μg) *in vivo* (Fig. 4; median value of 2215 RLU/mg for minicircle DNA and 1249 RLU/mg for plasmid DNA).

**Figure 5:**
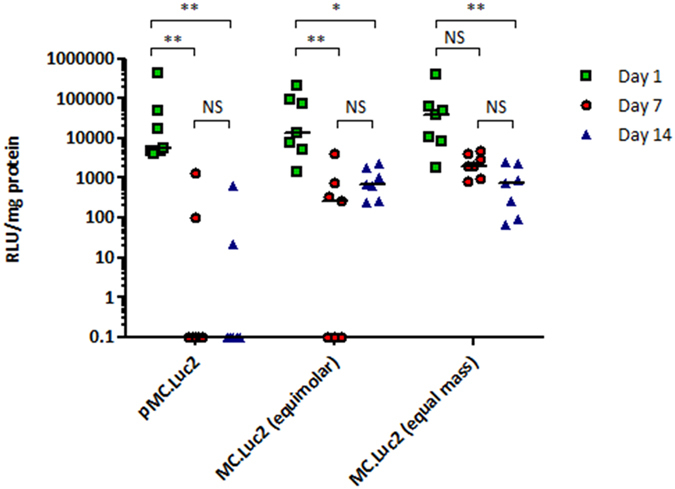
Transgene expression following *in vivo* minicircle and plasmid DNA transfections. Luciferase activity at 1, 7 and 14 days following a single dose of LED-1 complexes containing pMC.Luc2 plasmid DNA or MC.Luc2 minicircle DNA at either equimolar or equal mass quantities delivered by oropharyngeal instillation of CD1 mice. NS, not significant; *P < 0.05; **P < 0.01; Kruskal-Wallis test followed by Dunn’s post-test analysis to assess significance. Values are background subtracted and bar represents median RLU/mg.

We then assessed transgene persistence following a single dose of LED-1 complexes formulated with plasmid DNA (pMC.Luc2) or minicircle DNA (MC.Luc2). Luciferase assays of lung extracts were performed at 1, 7 and 14 days following a single dose of luciferase gene delivery. The median level of luciferase activity was 5674.9 RLU/mg 1 day following oropharyngeal instillation of 16.7 μg of pMC.Luc2 but at 7 and 14 days luciferase activity was undetectable in 5/7 mice (Fig. 5; p < 0.01). In contrast, following instillation of 7.1 μg (equimolar) and 16.7 μg (equal mass) of MC.Luc2 luciferase expression remained detectable in all mice at 14 days following transfection (Fig. 5) although there was a significant loss of luciferase expression between 1 day and 14 days following transfection (Fig. 5; for MC.Luc2 (equimolar) median value of 13676.2 RLU/mg at day 1 falls to 631.0 RLU/mg at ay 14 p < 0.05; for MC.Luc2 (equal mass) median value of 38030.7 RLU/mg at day 1 falls to 719.6 RLU/mg at ay 14 p < 0.01). Thus, equal weight or equimolar doses of minicircle DNA with the same cDNA and regulatory elements gives more persistent transgene expression than plasmid DNA.

### Minicircle toxicity compared to plasmid DNA formulations

**Figure 6:**
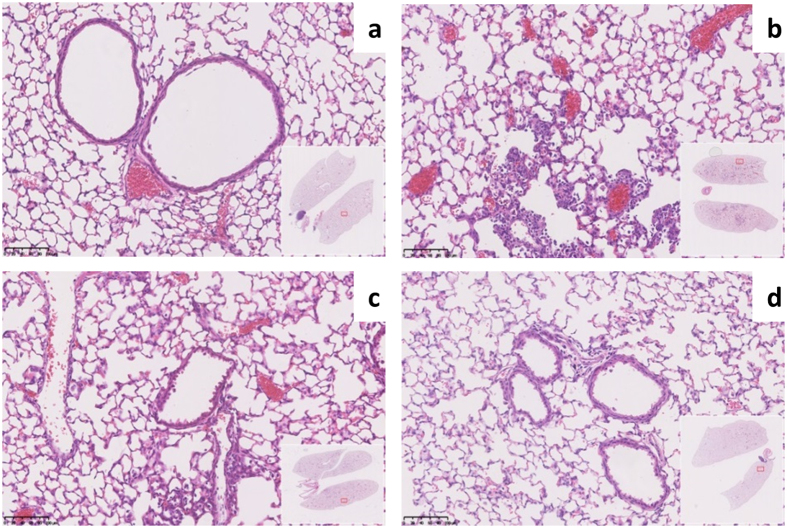
H&E staining of mouse lungs following *in vivo* minicircle and plasmid DNA transfections. H&E stained CD1 mice lung sections following oropharyngeal instillations of (**a**) water as a vehicle control, LED-1 vectors containing (**b**) pMC.Luc2, (**c**) MC.Luc2 at an equal mass to pMC.Luc2 and (**d**) MC.Luc2 equimolar to pMC.Luc2. Scale bars are 100 μm.

LED-1 nanocomplexes formulated with minicircle DNA contain less DNA, as well as liposome and peptide, than complexes formulated with equimolar amounts of plasmid DNA. We hypothesised the lower mass of DNA may translate to reduced toxicity *in vivo*. 24 hours after LED-1 transfections with luciferase encoding pMC.Luc2 or MC.Luc2 lungs were fixed by perfusion with 4% PFA and processed for H&E staining. Lungs of mice receiving the vehicle control, MilliQ water, showed no signs of inflammation or cellular infiltration by histology (Fig. 6a). In contrast, the lungs of mice transfected with plasmid or minicircle DNA showed evidence of patchy, mild peribronchial inflammation which was predominantly of a monocyte/macrophage nature. However, there were no clear differences when comparing plasmid and minicircle transfections (Fig. 6b–d).

**Figure 7:**
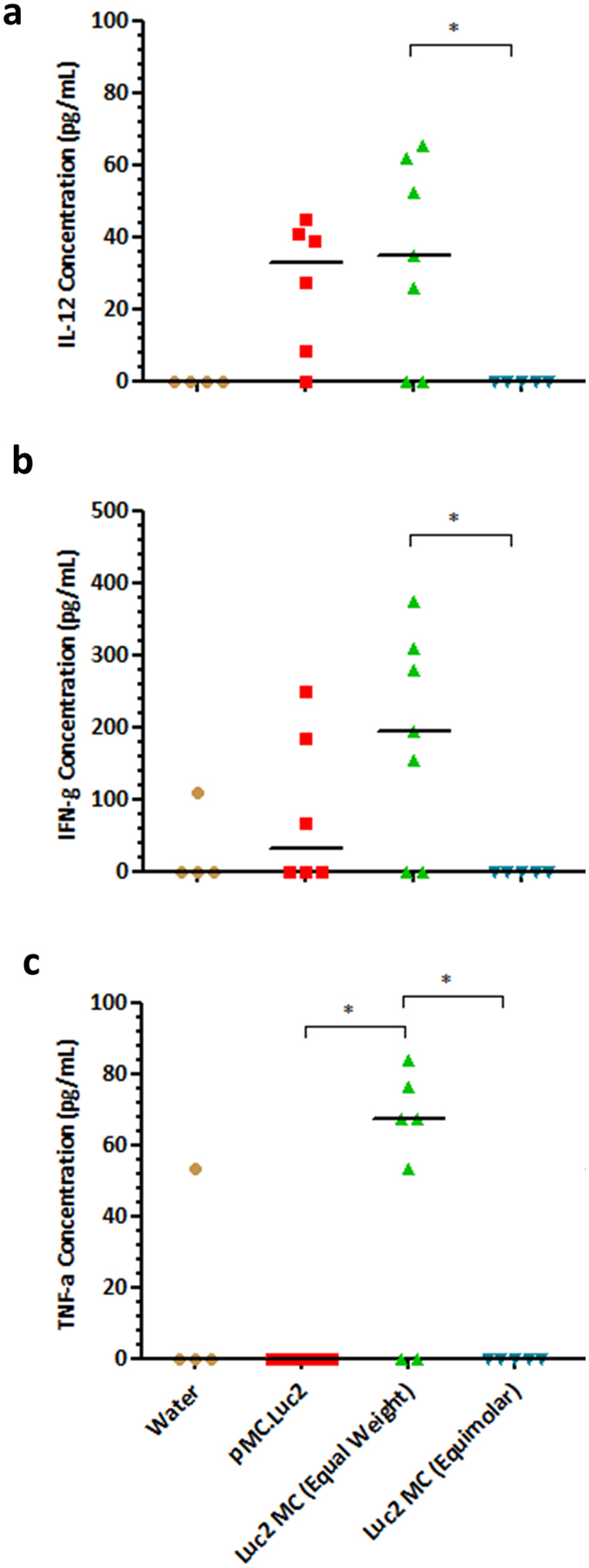
Cytokine response in BALF following *in vivo* minicircle and plasmid DNA transfections. Concentration of (**a**) IL-12, (**b**) IFN-γ and (**c**) TNF-α cytokines in BALF following instillation of LED-1 vectors containing luciferase encoding minicircle or plasmid DNA. Water was used as a vehicle control. *P < 0.05; Kruskal-Wallis test followed by Dunn’s post-test used to assess significance. Bar represents median.

Toxicity was further assessed by quantification of cytokine release. Bronchoalveolar lavage fluid (BALF) was obtained 24 hours following transfections with pMC.Luc2 plasmid or MC.Luc2 minicircles. Levels of IL-12 and IFN-γ were similar in BALF from mice receiving LED-1 vectors containing 16.7 μg of plasmid or minicircle DNA (Fig. 7a–b). Transfections with plasmid DNA or equimolar amount of minicircle yielded no detectable TNF-α expression in BALF which contrasted with a median of 67.22pg/mL following an equal mass minicircle transfection (Fig. 7c; p < 0.05). We also assessed levels of GM-CSF, IL-1β, IL-2, IL-4, IL-5, IL-6 and IL-10 and all were undetectable in all BALF samples. Encouragingly, all cytokines were undetectable in BALF from lungs of mice transfected with 7.1 μg of minicircle DNA (equimolar).

## Discussion

Gene delivery to the airway epithelium has been investigated for a number of diseases with most progress being made in the context of CF lung disease. We have previously shown that a non-viral formulation of a cationic DHDTMA/DOPE liposome and a cationic targeting peptide complexed with plasmid DNA efficiently transfected the airway epithelium of mice and pigs^18^,^21^,^22^. The peptide component is important for the formation of small and stable nanoparticles and receptor targeting whilst the liposome component is vital for endosomal escape to prevent degradation of the DNA payload^17^,^20^.

In this study we have shown that LED-1 formulations containing minicircle DNA can provide greater and more persistent transgene expression which could increase the prospect of successful gene therapy for airway diseases. Irrespective of whether LED-1, Lipofectamine 2000 or 25 kDa branched PEI was used as the transfection reagent, we have demonstrated that minicircle DNA enhanced transgene expression in airway cells *in vitro*. Other reports using minicircle DNA in a variety of human cell lines similarly showed 2–3 fold higher levels of transgene expression with minicircle DNA compared to plasmid DNA^45^. Our eGFP expression studies showed that both the percentage of transfected cells and the amount of protein produced was higher when using minicircle DNA. As in previous studies^45^, enhanced transgene expression was seen when transfecting equimolar amounts of minicircle and plasmid DNA, confirmed by qPCR analysis, highlighting that enhanced expression from minicircle DNA was not due to an increased number of transgene expression cassettes. Previous reports have demonstrated an inverse correlation between plasmid DNA size and transfection efficiency, despite equimolar amounts of DNA being transfected^46^,^47^. More recent studies have implicated the length of DNA sequences outside of the transgene expression cassette, irrespective of sequence origin, in lowering levels of transgene expression and persistence^48^. Minicircles have very little extragenic sequences so explaining their superiority over plasmid DNA which have sizeable bacterial backbones (~4 kb in this study).

Importantly for PCD gene therapy, we have also shown enhanced transgene expression with minicircle DNA when transfecting equimolar amounts of the large 14 kb *DNAH5* cDNA. Here the plasmid DNA was 19.1 kb compared to 15.1 kb for the minicircle. To our knowledge this is the largest minicircle produced to date and provides evidence that the attB-attP recombination system for minicircle production^30^ can accommodate large DNA sequences.

In contrast to *in vitro* studies, no significant differences in the initial levels of transgene expression were seen here *in vivo* comparing equimolar amounts of plasmid and minicircle DNA transfections. These findings are consistent with previous observations in the mouse liver^31^,^49^ where the major advantage of minicircle DNA was more persistent transgene expression. Similar to previous studies we found transgene expression from plasmid and minicircle DNA fell at 7 days post-transfection but the fall was greater with plasmid DNA and transgene expression from minicircles appeared to have stabilised^49^ with no significant differences found when comparing transgene expression 7 and 14 days post-transfection. Luciferase activity was undetectable, in most cases, at 7 and 14 days following plasmid DNA transfection in contrast to minicircle DNA where luciferase activity was detectable in all mice 14 days post-transfection. As such, whilst transgene expression levels were similar 24 hours following transfection, minicircle transfections gave more prolonged expression. This persistent transgene expression could have resulted from a greater loss of plasmid DNA, increased silencing of plasmid DNA or a mixture of both. Viecelli *et al.*^33^, and Chen *et al.*^31^, found no difference in the number of plasmid and minicircle DNA molecules in the liver despite more persistent transgene expression using minicircle DNA. Further studies highlighted extragenic DNA sequences above 500bp can induce transgene silencing^48^ in the liver which was due to chromatin-linked silencing of transcription^50^. Whether this mechanism explains the more persistent transgene expression we observed in mouse airways needs further investigation.

Lung transfections with the same mass of minicircle or plasmid DNA showed an approximately 6.5-fold increase in expression at 24 hours post-transfection with minicircle DNA. The MC.Luc2 minicircle at 2.9 kb used in this study was less than half (42.8%) the size of the pMC.Luc2 plasmid at 6.9 kb, therefore, in equal mass transfections there are 2.3-times more copies of the luciferase transgene in the minicircle transfection compared to the plasmid. The increased copies of luciferase transgene with minicircle transfection could explain, at least in part, the enhanced levels of transgene expression observed. However, it is of note that transgene expression was 6.5-fold greater with minicircle compared to plasmid DNA but only 2.3-times more luciferase transgenes were transfected with minicircle DNA. As such, it is unlikely that the 2.3-fold increase in the number of transgene expression cassettes fully explains the significantly higher increase in luciferase expression observed with equal mass minicircle transfections.

The LED-1 vector is formulated at a constant 0.75:4:1 mass ratio of liposome: peptide: DNA therefore in equimolar transfections the dose of all three components is lower when using minicircles. As such, we hypothesised that this lower dose would result in reduced toxicity. To test this hypothesis we assessed toxicity by histological analysis and cytokine levels in BALF. Histological analysis showed foci of mild inflammation in all transfected mice, consistent with our previous studies using the LED-1 vector^18^, and we found no obvious differences in severity comparing plasmid and minicircle DNA transfections. This patchy and localised inflammation has been associated with the bolus delivery of vector causing a transient and acute inflammatory response that can be circumvented by delivery of nebulised formulations^51^. Indeed when nebulisation was used with the LED-1 vector we found no inflammation in treated pigs^22^ in contrast to when a bolus of vector was administered^52^. However, to ensure accurate dosing a bolus of vector was instilled in this study.

In assessing cytokine levels in BALF we found in transfections with equimolar amounts of minicircle and plasmid DNA that 10 different cytokines 24 hours following transfection were all undetectable in BALF of mice transfected with minicircle. In contrast, levels of IFN-γ and IL-12 following plasmid DNA transfections were elevated compared to minicircle transfections and water controls supporting our hypothesis.

There were no significant differences in IFN-γ and IL-12 levels in BALF when equal masses (16.7 μg) of minicircle and plasmid DNA were transfected. However, TNF-α was elevated following transfection with minicircle but not plasmid DNA. TNF-α is produced by activated macrophages, monocytes, neutrophils and infected airway epithelial cells and is involved in regulating the production of other pro-inflammatory cytokines^53^,^54^,^55^. Resident macrophages in the lung engulf potential pathogens and gene delivery vectors and initiate an innate immune response. Immune responses following non-viral gene delivery can typically be attributed to the lipid or peptide based vector components^51^, bacterial DNA sequences as these are rich in unmethylated CpG-motifs^56^,^57^,^58^,^59^,^60^ and/or antigens from the transgene product, the principle behind DNA vaccines. With regards to the vector components the amounts of liposome, peptide and DNA delivered were the same in transfections with equal masses of plasmid and minicircle DNA, so these would not have contributed to the elevated levels of TNF-α. Also, the minicircle, in contrast to the plasmid, has no plasmid backbone so this also would not explain the elevated TNF-α levels following equal mass transfection of plasmid and minicircle DNA. It is possible, therefore, that the elevated TNF-α release with minicircle DNA was due to the over 6-fold increase in the amount of foreign luciferase protein expressed. The development of an immune response to transgene products has been reported previously following airway gene delivery^51^. Whilst the LED-1 vector primarily transfects airway epithelial cells, transgene expression in macrophages is also seen^18^ and greater and more persistent transgene expression would be expected to lead to a more robust immune response. Indeed minicircles encoding antigens can be more immunogenic than plasmids and provided more effective immunisation in mice^61^.

The minicircle DNA used in this study has potential for optimisation by further minimising the number of CpG-motifs present which has been shown to reduce the numbers of neutrophils, lung macrophages and pro-inflammatory cytokines, including IL-12, IFN-γ and TNF-α in BALF, following airway gene transfer in mice^25^,^26^,^27^. In addition, the exchange of the human elongation factor 1- alpha (EF1α) promoter for human polyubiquitin C (UbC) promoter could provide more enhanced and sustained transgene expression^24^ bringing the prospect of successful airway gene therapy for human diseases ever closer.

In summary, we show that minicircles provide prolonged transgene expression *in vivo* and, relative to plasmid DNA, can be used at equimolar quantities to significantly reduce toxicity and immunogenicity or used at equal mass quantities to significantly enhance transgene expression. Our findings thus have clear implications for gene therapy of CF, PCD and of other airway disorders where plasmid DNA transfections, despite optimisation, have so far proven inefficient in clinical trials^62^.

## Additional Information

**How to cite this article**: Munye, M. M. *et al.* Minicircle DNA Provides Enhanced and Prolonged Transgene Expression Following Airway Gene Transfer. *Sci. Rep.* **6**, 23125; doi: 10.1038/srep23125 (2016).

## Supplementary Material

Supplementary Information
