# Supplementary material for: Minicircle DNA Provides Enhanced and Prolonged Transgene Expression Following Airway Gene Transfer
Source: Sci Rep. 2016 Mar 15;6:23125. doi: 10.1038/srep23125 (PMC4792149; doi:10.1038/srep23125)
Supplement: Supplementary Information [file srep23125-s1.pdf]

Minicircle DNA Provides Enhanced and Prolonged Transgene Expression Following Airway Gene Transfer

Munye MM*[1]; Tagalakis AD*[1]; Barnes JL[2]; Brown RE[3]; McAnulty, RJ[2]; Howe, SJ[1]; Hart SL[1]

[1] UCL Institute of Child Health, 30 Guilford Street, London, WC1N 1EH, United Kingdom

[2] UCL Respiratory Centre for Inflammation and Tissue Repair, 5 University Street, London, WC1E 6JF, United Kingdom

[3] UCL MRC Laboratory for Molecular Cell Biology, Gower Street, London WC1E 6BT, United Kingdom

* These authors contributed equally to this work.

Correspondence should be addressed to S.L.H. (s.hart@ucl.ac.uk)

UCL Institute of Child Health, 30 Guilford Street, London, WC1N 1EH, United Kingdom

SUPPLEMENTARY MATERIALS & METHODS

Materials

Peptide K_{16}GACSERSMNFCG[1] was synthesised by China Peptide Co., Ltd (Shanghai, China), and dissolved in endotoxin-free water (Sigma-Aldrich, Dorset, UK) to 10 mg/mL. Liposome consisted of 1-Propanaminium, *N*,*N*,*N*-trimethyl-2,3-bis(11*Z*-hexadecenyloxy)-chloride (DHDTMA chloride; Avanti Polar Lipids; Alabama, USA) with dioleoylphosphatidylethanolamine (DOPE; Avanti Polar Lipids; Alabama, USA) formulated at a 1:1 weight ratio and dissolved in sterile water to give a 2 mg/mL liposome suspension.

Minicircle production

Minicircle DNA (MC) was produced from the minicircle producer plasmid (pMC) as previously described[2] and agarose gel electrophoresis used to assess relative purity of minicircle and plasmid DNA preparations.

Cell culture

16HBE14o- cells (Dieter Gruenert, San Francisco[3]) were cultured in complete media consisting of Eagle's Minimal Essential Medium (MEM; Sigma-Aldrich, Dorset, UK) supplemented with 10% (v/v) foetal bovine serum, 100 U/mL penicillin, 100 mg/mL streptomycin, 2 mmol/l L-glutamine and incubated in humidified 5% CO_2 at 37°C.

In vitro transfections

Cells were seeded in black 96-well plates (Fisher Scientific UK, Leicestershire, UK) at a density of 20,000 cells per well and incubated in humidified 5% CO_2 at 37°C for 24 hours. For luciferase assay experiments, cells were seeded in. All complexes were prepared in OptiMEM (Life Technologies, Paisley, UK). Lipofectamine 2000 complexes were prepared

according to the manufacturer's instruction at a Lipofectamine 2000:DNA ratio of 4:1 and complexes of 25kDa branched PEI were formulated at a N/P ratio of 10:1 as described previously[4]. Complexes were formulated to a plasmid/minicircle DNA concentration of 10µg/mL and diluted to the appropriate concentrations before incubation with cells in humidified 5% CO_2 at 37^oC for 24hours.

Luciferase assay following *in vitro* transfection

Cells were washed with 1x PBS 24 hours following transfection and lysed with 20µL Reporter Lysis Buffer (Promega, Southampton, UK) for 20 minutes at 4^oC then -80^oC for at least 30 minutes followed by thawing at room temperature. Luciferase activity was assessed using the Luciferase Assay System (Promega, Southampton, UK) on a FLUOstar Optima plate reader (BMG Labtech, Aylesbury, UK). The results were standardised for protein content using the Bradford protein assay (Bio-Rad, Hertfordshire, UK) and expressed as relative luminescence units per mg of protein (RLU/mg). Assessment of luciferase activity at 24 hours was chosen as firefly luciferase is known to have a short half-life (3h)[5] and expression following lung gene delivery *in vivo* has been shown to be maximal at 24 hours[6,7].

Measurement of complex size and charge

LED-1 nanoparticles were formulated to a plasmid/minicircle DNA concentration of 10µg/mL in water and incubated at room temperature for 30 minutes to allow complex formation. Complexes were then diluted in 1mL dH2O to a final concentration of 2µg/mL and hydrodynamic size and surface charge (ζ potential) were measured using a Malvern Nano ZS (Malvern, Worcestershire, UK) as previously described[8].

Statistical analysis

Data are mean values ± standard error of the mean (SEM) for *in vitro* studies and median with all data points shown for *in vivo* studies. Student's t-test was used to assess statistical significance. Significance levels were set at $p<0.05$. * $=p<0.05$; ** $=p<0.01$; *** $=p<0.001$; NS = not significant.

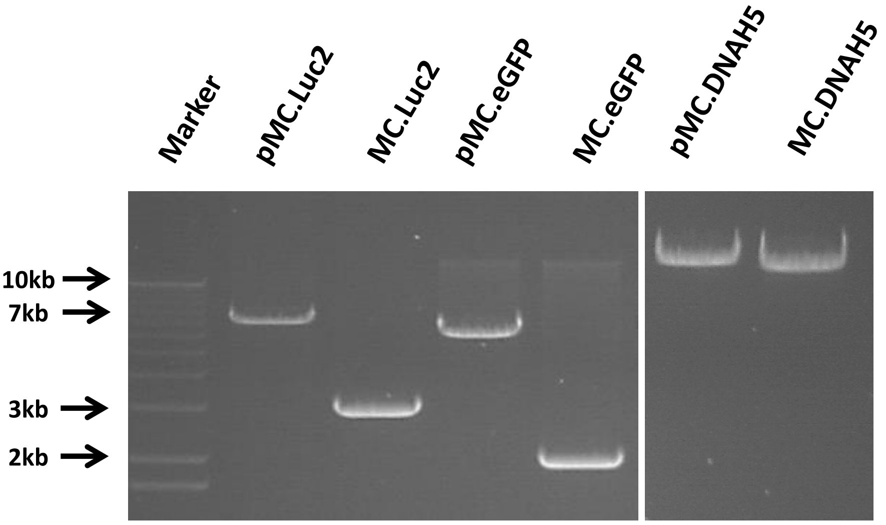

Figure S1. Purity of minicircle DNA produced in vivo from plasmid constructs. 500ng of plasmid (pMC) and minicircle (MC) DNA were digested with single cutting restriction enzymes (*SalI* for DNAH5 and Luc2 and *EcorV* for eGFP) thus linearising the DNA and run on a 1% agarose gel.

OptiMEM

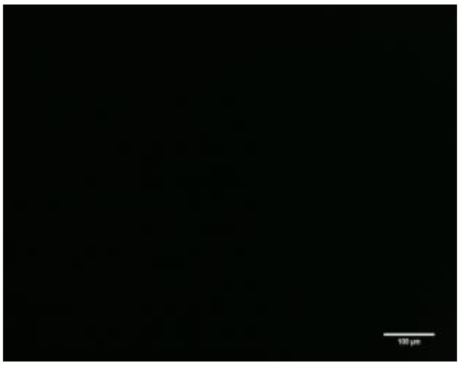

pMC.eGFP (77.91 fmol)

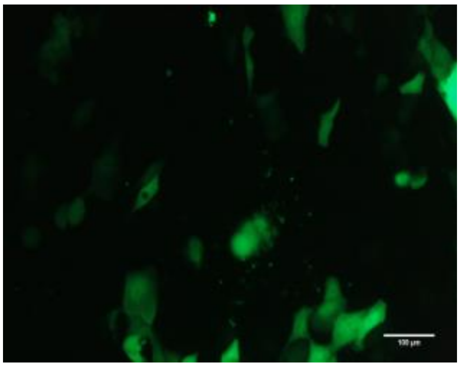

MC.eGFP (77.91 fmol)

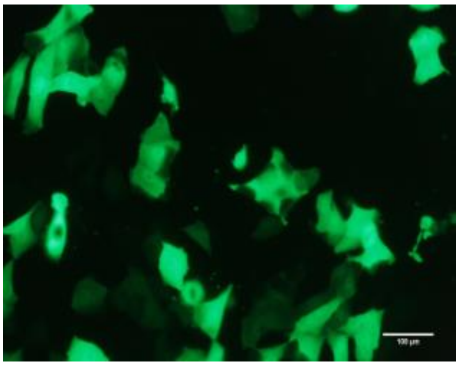

Figure S2. eGFP expression following *in vitro* transfections. 16HBE14o- cells were transfected with minicircle DNA or plasmid DNA carrying the eGFP gene and representative fluorescence microscopy images captured. Scale bars are 100µm.

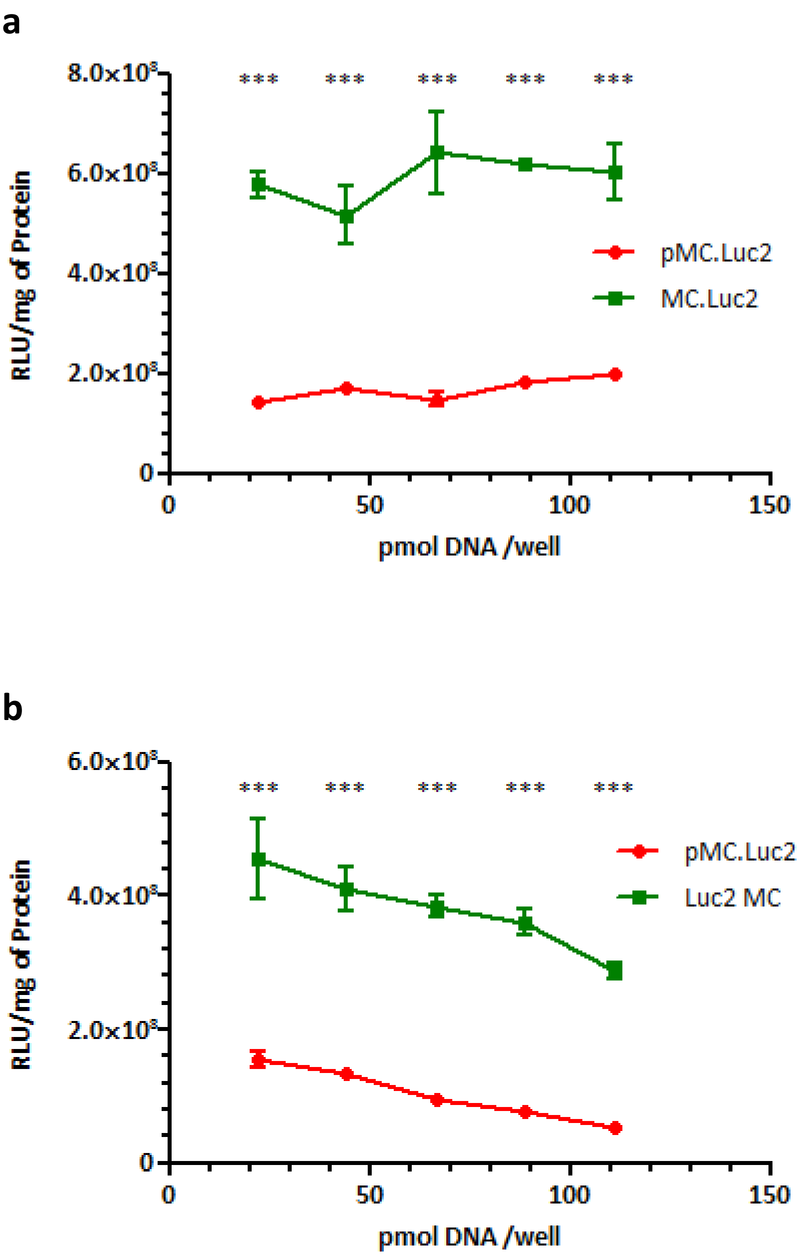

Figure S3. Luciferase expression following *in vitro* transfections. 16HBE14o- cells were transfected with plasmid or minicircle DNA carrying the firefly luciferase gene using a) Lipofectamine 2000 and b) PEI. ***$P<0.001$; Student's t-test used to assess significance. Values are background subtracted and displayed as mean ±SEM.

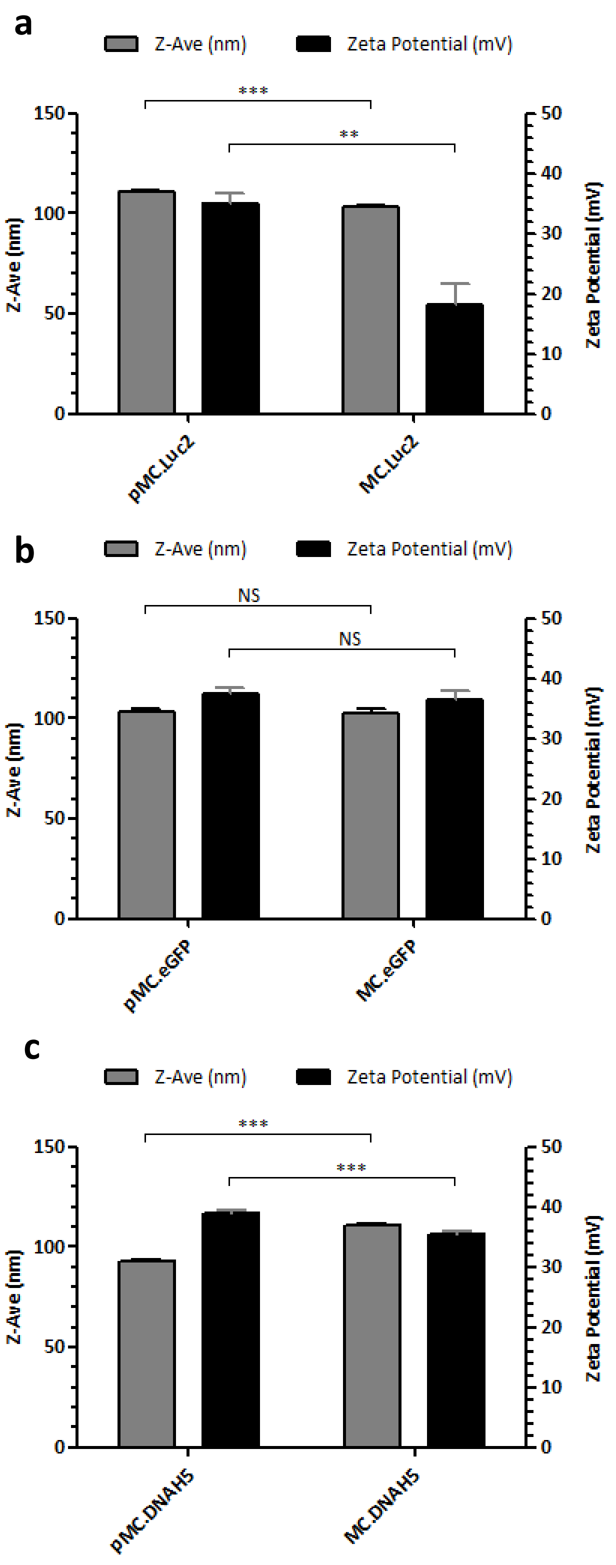

Figure S4. Bio-physical properties of LED-1 complexes formulated with plasmid and minicircle DNA. LED-1 complexes were formulated with plasmid or minicircle DNA coding for a) firefly luciferase, b) eGFP or c) DNAH5 and nanoparticle size and charge was assessed. NS, not significant, **P<0.01, ***P<0.001; Student's t-test used to assess significance. Values are mean ±SEM.

SUPPLEMENTARY MATERIAL

Figure S1. Purity of minicircle DNA produced in vivo from plasmid constructs. 500ng of plasmid (pMC) and minicircle (MC) DNA were digested with single cutting restriction enzymes (*SalI* for DNAH5 and Luc2 and *EcorV* for eGFP) thus linearising the DNA and run on a 1% agarose gel.

Figure S2. eGFP expression following *in vitro* transfections. 16HBE14o- cells were transfected with minicircle DNA or plasmid DNA carrying the eGFP gene and representative fluorescence microscopy images captured. Scale bars are 100µm.

Figure S3. Luciferase expression following in vitro transfections. 16HBE14o- cells were transfected with plasmid or minicircle DNA carrying the firefly luciferase gene using a) Lipofectamine 2000 and b) PEI. ***P<0.001; Student's t-test used to assess significance. Values are background subtracted and displayed as mean ±SEM.

Figure S4. Bio-physical properties of LED-1 complexes formulated with plasmid and minicircle DNA. LED-1 complexes were formulated with plasmid or minicircle DNA coding for a) firefly luciferase, b) eGFP or c) DNAH5 and nanoparticle size and charge was assessed. NS, not significant, **P<0.01, ***P<0.001; Student's t-test used to assess significance. Values are mean ±SEM.

REFERENCES

1 Writer, M. J. *et al.* Targeted gene delivery to human airway epithelial cells with synthetic vectors incorporating novel targeting peptides selected by phage display. *J. Drug Target.* **12**, 185-193 (2004).

2 Kay, M. A., He, C. Y. & Chen, Z. Y. A robust system for production of minicircle DNA vectors. *Nat. Biotechnol.* **28**, 1287-U1296 (2010).

3 Cozens, A. L. *et al.* CFTR expression and chloride secretion in polarized immortal human bronchial epithelial cells. *Am. J. Respir. Cell Mol. Biol.* **10**, 38-47 (1994).

4 Tagalakis, A. D. *et al.* A receptor-targeted nanocomplex vector system optimized for respiratory gene transfer. *Mol. Ther.* **16**, 907-915 (2008).

5 Thompson, J. F., Hayes, L. S. & Lloyd, D. B. Modulation of Firefly Luciferase Stability and Impact on Studies of Gene-Regulation. *Gene* **103**, 171-177 (1991).

6 Rudolph, C., Lausier, J., Naundorf, S., Muller, R. H. & Rosenecker, J. In vivo gene delivery to the lung using polyethylenimine and fractured polyamidoamine dendrimers. *J. Gene Med.* **2**, 269-278 (2000).

7 Gautam, A., Densmore, C. L., Xu, B. & Waldrep, J. C. Enhanced gene expression in mouse lung after PEI-DNA aerosol delivery. *Mol. Ther.* **2**, 63-70 (2000).

8 Jenkins, R. G. *et al.* Formation of LID vector complexes in water alters physicochemical properties and enhances pulmonary gene expression in vivo. *Gene Ther.* **10**, 1026-1034 (2003).
